# Therapeutic relationship and virtual setting during the COVID 19 emergency

**DOI:** 10.1192/j.eurpsy.2021.926

**Published:** 2021-08-13

**Authors:** S. Cerino

**Affiliations:** Sphere, ECOS - EU, Massa Martana, Italy

**Keywords:** virtual setting, psychotherapy, covid 19 emergency

## Abstract

**Introduction:**

During the COVID 19 epidemic, the isolation helped virtual psychoterapy sessions, not to break up therapeutic relationship in critical moments.

**Objectives:**

This paper points out the traditional setting modification and how the interpersonal relationship can affect the therapeutic dynamics.

**Methods:**

The experience could support the possibility to design adequate plans to test possible relational potentiality/prospect to respond to the pandemic emergency. The computer screen represents a very important new and rich element as “Skype” seems to have been the most used remote support. The screen plays a filter and separation function but physically represents the related presence in a shared timeframe. It is also a “mutual mirror”, reflecting the exclusive duality and resending to “different” space and time where the therapeutic relationship acts.

**Results:**

In this way the “analysis room” loses its physical feature to move towards a new dimension where the subjective experience are communicated/lived/re-elaborated by the mean of shared visual, modifyng the codified space of a traditional setting.

**Conclusions:**

The screen is not only a mere vehicle of verbal communication, but fully gets in “hic et nunc” in space relationship assuming however an allegoric value, that, in the individual subjective, could go really beyond its “simple” and usual technological function.

